# Prediction Models Discriminating between Nonlocomotive and Locomotive Activities in Children Using a Triaxial Accelerometer with a Gravity-removal Physical Activity Classification Algorithm

**DOI:** 10.1371/journal.pone.0094940

**Published:** 2014-04-22

**Authors:** Yuki Hikihara, Chiaki Tanaka, Yoshitake Oshima, Kazunori Ohkawara, Kazuko Ishikawa-Takata, Shigeho Tanaka

**Affiliations:** 1 Faculty of Engineering, Chiba Institute of Technology, Chiba, Japan; 2 Division of Integrated Sciences, J. F. Oberlin University, Tokyo, Japan; 3 Faculty of Service Industries, University of Marketing and Distribution Science, Hyogo, Japan; 4 Faculty of Informatics and Engineering, University of Electro-Communications, Tokyo Japan; 5 Department of Nutritional Education, National Institute of Health and Nutrition, Tokyo Japan; 6 Department of Nutritional Science, National Institute of Health and Nutrition, Tokyo Japan; University of California, Irvine, United States of America

## Abstract

The aims of our study were to examine whether a gravity-removal physical activity classification algorithm (GRPACA) is applicable for discrimination between nonlocomotive and locomotive activities for various physical activities (PAs) of children and to prove that this approach improves the estimation accuracy of a prediction model for children using an accelerometer. Japanese children (42 boys and 26 girls) attending primary school were invited to participate in this study. We used a triaxial accelerometer with a sampling interval of 32 Hz and within a measurement range of ±6 G. Participants were asked to perform 6 nonlocomotive and 5 locomotive activities. We measured raw synthetic acceleration with the triaxial accelerometer and monitored oxygen consumption and carbon dioxide production during each activity with the Douglas bag method. In addition, the resting metabolic rate (RMR) was measured with the subject sitting on a chair to calculate metabolic equivalents (METs). When the ratio of unfiltered synthetic acceleration (USA) and filtered synthetic acceleration (FSA) was 1.12, the rate of correct discrimination between nonlocomotive and locomotive activities was excellent, at 99.1% on average. As a result, a strong linear relationship was found for both nonlocomotive (METs = 0.013×synthetic acceleration +1.220, R^2^ = 0.772) and locomotive (METs = 0.005×synthetic acceleration +0.944, R^2^ = 0.880) activities, except for climbing down and up. The mean differences between the values predicted by our model and measured METs were −0.50 to 0.23 for moderate to vigorous intensity (>3.5 METs) PAs like running, ball throwing and washing the floor, which were regarded as unpredictable PAs. In addition, the difference was within 0.25 METs for sedentary to mild moderate PAs (<3.5 METs). Our specific calibration model that discriminates between nonlocomotive and locomotive activities for children can be useful to evaluate the sedentary to vigorous PAs intensity of both nonlocomotive and locomotive activities.

## Introduction

Much research has shown that there is a positive relationship between physical activity (PA) and both physical and mental health outcomes in children [1], [2]. It is currently recommended that children should be engaged in moderate to vigorous intensity physical activity (MVPA) for at least 60 minutes each day [2], [3]. Therefore, it is important to evaluate the exact PA intensity needed to improve and maintain an individual’s physical and emotional health.

Estimation of PA in children is particularly difficult, since children show PA of varying intensity with short duration [4], [5]. PA is generally estimated in units of activity energy expenditure or time engaged in MVPA. To date, a variety of methods has been used to measure PA in children and adolescents. Although questionnaires and self-report activity diaries are effective methods in population-based research, they have the limitations of being less valid and reliable, particularly in children [6].

Accelerometers have recently come into wide use as monitors of PA. Accelerometers have the advantages of being objective, cost effective, and minimally invasive [7]. Previous studies proposed prediction models of metabolic equivalents (METs) for children with accelerometers [8]–[16]. These prediction models were based on the linear relationship between oxygen uptake and acceleration during several typical activities that reflect daily lifestyle activities of children. Typically, the selected activities are of low (<3 METs), moderate (3–5.9 METs) and vigorous intensity (≥6 METs). The slope and intercept of a predictive model of locomotive activities, such as walking and running, are different from those of nonlocomotive activities, like playing games, cleaning, playing with blocks, tossing a ball, and aerobic dance [9]–[11], [15]. Interestingly, Crouter et al. [17] proposed a new child-specific, two-regression model (2 RM), which is able to discriminate between locomotive activities, such as continuous walking or jogging, and nonlocomotive activities, including lifestyle activity, on the basis of the variability in the accelerometer count. The estimation accuracy of PA with the 2 RM depends on the sensitivity of discriminating between locomotive and nonlocomotive activities [18], [19]. We also suggested a new calibration model that could discriminate locomotive activities from nonlocomotive activities in adults with a triaxial accelerometer based on the ratio of raw synthetic acceleration to filtered synthetic acceleration without gravity acceleration (gravity-removal physical activity classification algorithm [GRPACA]) [20], [21]. The rate of correct discrimination between nonlocomotive (household) and locomotive activities was 98.7% for 11 selected activities in adults [21].

Our initial aim was to examine whether the GRPACA is able to discriminate between locomotive and nonlocomotive activities for various PAs of children. Our second aim was to prove that this discrimination method improves the estimation accuracy of the prediction model for children using an accelerometer.

## Materials and Methods

### Participants

Healthy Japanese children (42 boys: 15 who were 6–9 years of age, and 27 who were 10–12 years of age and 26 girls: 14 who were 6–9 years of age, and 12 who were 10–12 years of age) attending primary school were invited to participate in this study via a public advertisement. None of the participants had physical impairments that could affect daily life activity or took any medications that could affect metabolism. All participants and parents were fully informed of the purpose of the study, and written informed consent was obtained from parents on behalf of the participants prior to the start of the study. This study was conducted according to the guidelines of the Declaration of Helsinki, and all procedures involving human participants were approved by the Ethical Committee of the National Institute of Health and Nutrition.

### Anthropometry

Body weight was measured to the nearest 0.1 kg with a digital balance (YL-65S, YAGAMI Inc., Nagoya, Japan), and height was measured on a stadiometer to the nearest 0.1 cm (YK-150D, YAGAMI Inc., Nagoya, Japan). Body mass index (kg/m^2^) was calculated as body weight divided by the square of body height.

### Procedures

To avoid diet-induced thermogenesis, the children visited the laboratory in the morning, three hours after breakfast. After the study protocol was fully explained, anthropometric measurements were taken. Next, participants were asked to rest for 30 minutes, and then the resting (in the seated position on a chair) metabolic rate was measured for 7 minutes; in children, it was measured while the child was viewing a video (e.g. Disney movie) to avoid fidgeting [22]. In addition, we asked the participants to put their hands on their thighs and to keep their feet on the floor during the measurement. Next, the children performed 11 PA for approximately 3 to 7 minutes, in addition to 3 minutes to obtain steady state (Table 1). First, nonlocomotive activities excluding throwing a ball were performed in order of PA intensity (lower to higher) with a few minutes of recovery between tasks, and after an approximately 10-minute break, climbing down and up activities were performed sequentially. Next, participants performed the throwing a ball activity. Locomotive activities were also conducted in order of PA intensity (lower to higher) with a few minutes of recovery between activities. All participants wore a triaxial accelerometer on the waist, tightly attached with a belt, during each activity. Before the experiment started, the accelerometers were synchronized using a wave clock for reference. Measurement of each activity began after a preliminary period that was needed to reach a steady-state condition with 3 minutes, based on our pilot study and previous studies [17], [20], [23]. The steady-state durations for climbing down and up were 2 minutes, because participants were moving to the implementation site on foot within a few minutes of the measurement of climbing down, and climbing up was performed after climbing down for 3 minutes. The energy expenditure (EE) of each activity was calculated from oxygen consumption (VO_2_) and carbon dioxide production (VCO_2_) with Weir’s equation [24]. To calculate the METs, we divided the EE during each activity by the measured value of the metabolic rate of the participant when seated on a chair.

**Table 1:** Description of performed calibration tasks.

Tasks	Content of activity	Intensity	Steady state(min)	Gathering expired gas (min)*
***Nonlocomotive***				
desk work	handwriting letters at a desk	light	3.0	4.0
Nintendo DS	playing Nintendo DS withsitting on the floor	light	3.0	3.0
sweeping up	sweeping floor (about 17 m^2^)while moving	light	3.0	3.0
clearing away	placing books from floor onto abookshelf	light	3.0	3.0
washing the floor	wiping down the floor with acloth in a squatting position	moderate	3.0	2.0
throwing a ball	playing catch with a large ballwith a partner	moderate	3.0	3.0
***Locomotive***				
climbing down	climbing down stairs accordingto a pace leader	moderate	2.0	1.0
climbing up	climbing up stairs accordingto a pace leader	vigorous	2.0	1.0
normal walking	normal walking speed accordingto a pace leader (60 m/min)on the ground	moderate	3.0	2.0
brisk walking	brisk walking speed accordingto a pace leader (80 m/min)on the ground	moderate	3.0	2.0
Jogging	jogging according to a paceleader (early grades:100 m/min,late grades: 120 m/min)	vigorous	3.0	2.0

*We collected expired gas for 1 to 4 min after steady state for 2 or 3 min.

### Triaxial Accelerometer

We used a triaxial accelerometer with 4 GB of memory (Omron Healthcare, Kyoto, Japan) consisting of a Micro electro-mechanical system-based accelerometer (LIS3LV02DQ; ST-Microelectronics), which responds to both acceleration due to movement and gravitational acceleration. The device for children measured 74 mm×46 mm×34 mm and weighed 60 g, including batteries. It was designed to detect accelerations in the vertical (x), anteroposterior (y), and mediolateral (z) axes with each activity at a rate of 32 Hz to 12-bit accuracy. The acceleration obtained from these specifications was passed through a high-pass filter with a cut-off of 0.7 Hz to exclude gravitational acceleration. We calculated the integral of the absolute value of the accelerometer value (synthetic acceleration), the square root of the sum of the square of the absolute acceleration from three axes (synthetic acceleration =  (X^2^+ Y^2^+ Z^2^)^0.5^). Finally, this device could record the synthetic acceleration of a 10-s epoch length within a measurement range of ±6 G and with a resolution of 3 mG. We analysed the acceleration data converted into a 10-s epoch length when collecting the expired gas for each activity. The reliability of this device was validated by the manufacturer, and is reported in technical reports (unpublished). The reliability test referred to the procedures of Japanese Industrial Standards (JIS7200∶1993), according to which a pedometer is validated with a vibration exciter.

### Indirect Calorimetry

Respiratory gas samples were analysed with the Douglas bag method, in which each participant was fitted with a facemask (No.09759, YAGAMI Inc., Nagoya, Japan) and breathed into a Douglas bag (No.35060, YAGAMI Inc., Nagoya, Japan). Participants performed calibration tasks person-to-person with an assistant who was holding the 50 L or 100 L-sized Douglas bag. The assistant opened a cock of the Douglas bag to collect the expired gas at the same time as the steady-state period finished, and then closed it when measurement finished without hindrance. The bag concentrations of oxygen and carbon dioxide were analyzed by a mass spectrometer (ARCO-1000; Arco System Inc., Kashiwa, Japan) that has recently come into wide use in several countries, in particular, Japan [20], [25]. The precision of the expired gas measurement was 0.02% for oxygen and 0.06% for carbon dioxide. The expired gas volume was measured with a certified dry gas meter (DC-5; Shinagawa Co., Ltd., Tokyo, Japan), the accuracy and precision of which were maintained within 1% of the coefficient of variation.

### Selection of Physical Activity for Calibration Models

We gathered information about the children’s habitual PA behavior at school and after school from direct interviews of another group of children and public reports of an education committee. Based on those sources of information, we selected 11 PAs for children that consisted of sedentary and light (<3 METs), moderate (3–5.9 METs), and vigorous activity (≥6 METs), according to the compendium of PAs [26], [27], to produce a calibration model.

### Discriminative Method

In our previous study, we reported an algorithm for the classification of nonlocomotive (household) and locomotive activities based on the ratio (e.g. cut-off value for adults, 1.16) of unfiltered synthetic acceleration (USA) to filtered synthetic acceleration (FSA) [21]. FSA was defined as the integrated acceleration ((X^2^+ Y^2^+ Z^2^)^0.5^) after the gravitational acceleration was removed from each dimensional acceleration (X, Y, Z) by passing through a second-order Butterworth high-pass filter [21]. Thus, the most important difference between USA and FSA is that FSA is not affected by a change in gravitational acceleration, while USA is. In adults, the rate of correct discrimination of nonlocomotive (e.g. household) from locomotive activities was 98.7% for 11 selected activities with the ratio (USA/FSA) [21]. Therefore, in this study, this discriminative procedure was applied to the children’s calibration model, and we aimed to determine a cut-off value for children.

### Statistical Analysis

Statistical analysis was performed with JMP version 8.0 for Windows (SAS Institute, Tokyo, Japan). All results are shown as mean ± standard deviation (SD). In the present study, we carried out multiple regression analysis with a stepwise method to examine the effects of weight, age and sex, and then analysis of covariance (ANCOVA) to assess the interaction (age×sex) on the measured METs prior to statistical analyses. The determination coefficient (R^2^) was used to evaluate the relationships between variables. One-way analysis of variance (ANOVA) was used to compare measured METs with predicted METs. Mean differences and limits of agreement between predicted METs and measured METs were determined by Bland and Altman plots [28]. Receiver-operating characteristic (ROC) curve analysis was applied to the acceleration data to assess the cut-off value for classification of nonlocomotive and locomotive activities. P<0.05 was considered statistically significant.

## Results

First, we divided the children into two groups: a development group and a cross-validation group. We randomly selected participants stratified by sex and age (6–9 yrs and 10–12 yrs). Characteristics of all children, the development group and the cross-validation group are shown in Table 2.

**Table 2:** Physical characteristics of the participants.

		Development group				Cross-validation group				Total participants			
		Boys (30)		Girls (18)		Boys (12)		Girls (8)		Boys (42)		Girls (26)	
		Mean	SD	Mean	SD	Mean	SD	Mean	SD	Mean	SD	Mean	SD
Age	(yrs)	10.0	1.8	9.2	2.1	10.1	1.5	8.8	1.2	10.0	1.7	9.0	1.8
Height	(cm)	140.2	12.4	134.9	14.2	141.1	7.5	131.4	10.6	140.5	11.5	134.4	12.6
Weight	(kg)	34.0	11.0	30.2	9.2	33.7	5.2	27.2	6.3	33.9	9.9	29.8	8.2
BMI	(kg/m^2^)	16.9	2.9	16.2	2.2	16.8	1.6	15.6	1.6	16.8	2.6	16.1	2.1

BMI; body mass index, SD; standard deviation.

**Figure 1:**
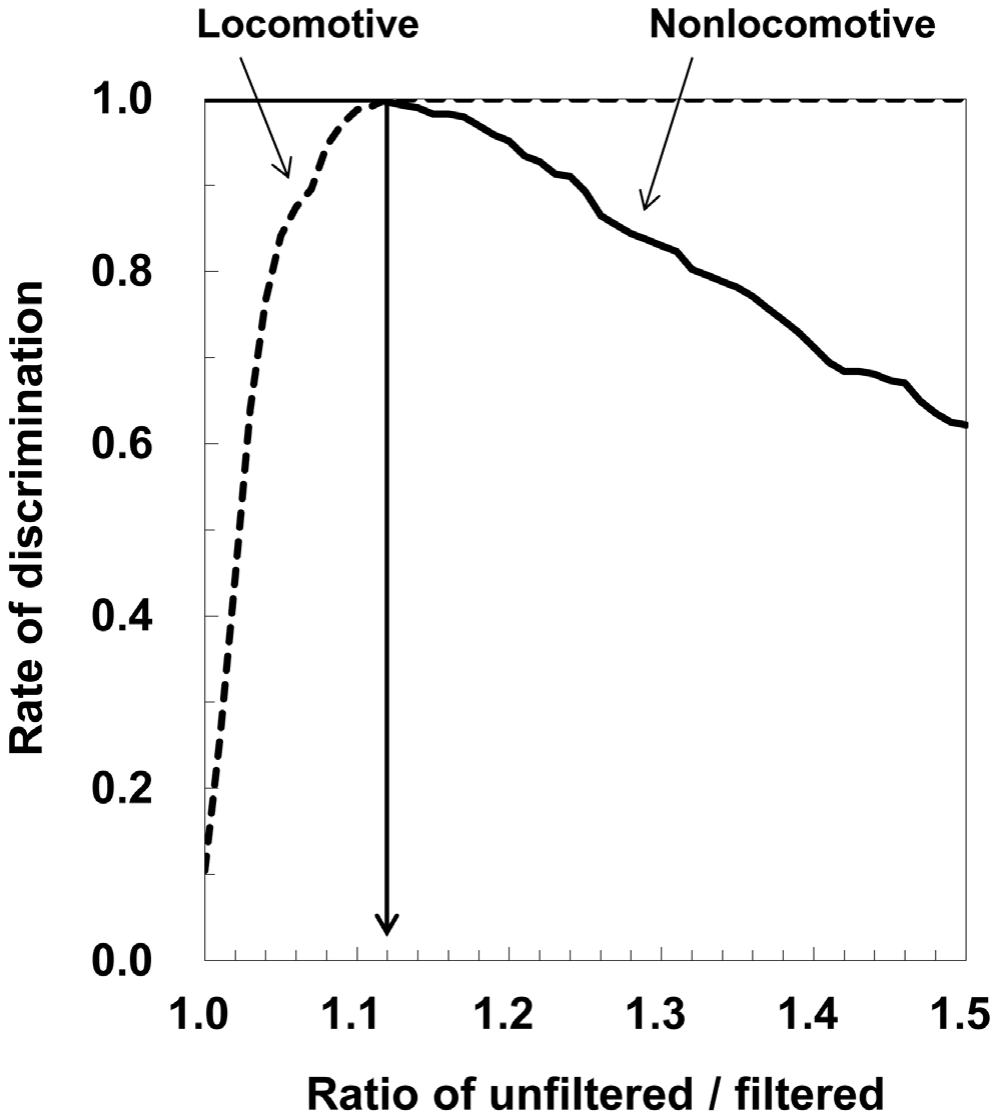
Probability of correctly detecting locomotive and nonlocomotive activities in the development group (n = 48).

**Figure 2:**
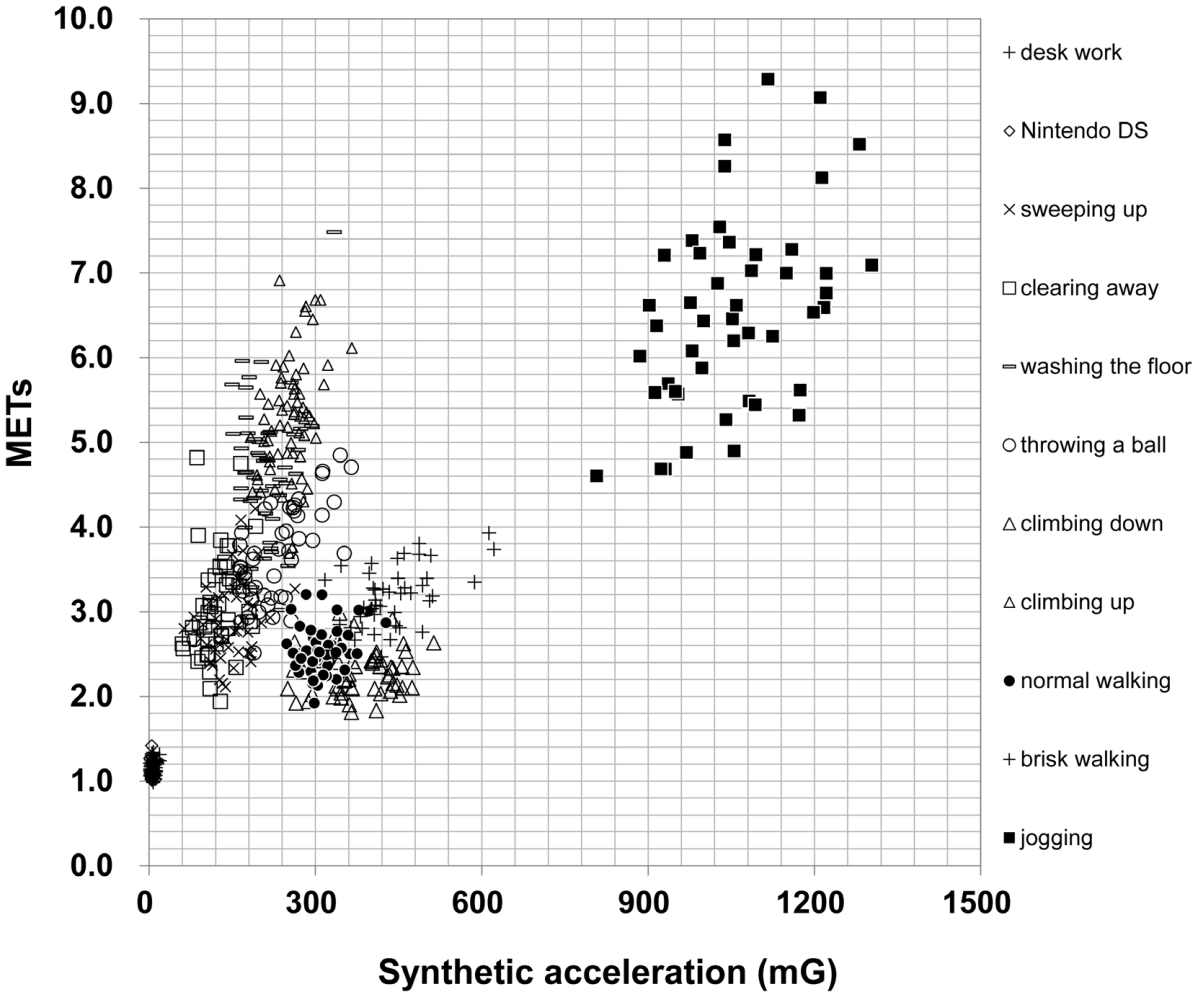
Relationship of synthetic acceleration to measured METs in nonlocomotive and locomotive activities in the development group (n = 48).

Discrimination with the ratio of USA/FSA provided the highest rate of correct discrimination, 99.8%, when the value of the ratio was 1.12 (Figure 1, Table 3). Therefore, we calculated the estimated METs through standard equations according to the results of discrimination with the ratio of 1.12, and then compared these values with the measured METs. The relationship between synthetic acceleration and METs is shown in Figure 2 (development group: n = 48). Plots of nonlocomotive activities were different from those of locomotive activities. In addition, plots of climbing down and up were located above and below the line, respectively. The linear regression equation is as follows:

**Table 3:** Rate of correct discrimination of nonlocomotive from locomotive activities.

Threshold	1.12*		1.13		1.14		1.15		1.16#	
	Development group (48)	Cross-validation group (20)	Development group (48)	Cross-validation group (20)	Development group (48)	Cross-validation group (20)	Development group (48)	Cross-validation group (20)	Development group (48)	Cross-validation group (20)
***Nonlocomotive***										
desk work	100.0	100.0	100.0	100.0	100.0	100.0	100.0	100.0	100.0	100.0
Nintendo DS	100.0	100.0	100.0	100.0	100.0	100.0	100.0	100.0	100.0	100.0
sweeping up	100.0	100.0	97.9	100.0	97.9	95.0	95.8	95.0	95.8	95.0
clearing away	100.0	100.0	100.0	100.0	100.0	100.0	100.0	100.0	100.0	100.0
washing the floor	100.0	100.0	100.0	100.0	100.0	100.0	100.0	100.0	100.0	100.0
throwing a ball	97.9	100.0	97.9	95.0	95.8	95.0	93.8	90.0	93.8	90.0
***Locomotive***										
climbing down	100.0	100.0	100.0	100.0	100.0	100.0	100.0	100.0	100.0	100.0
climbing up	100.0	90.0	100.0	95.0	100.0	95.0	100.0	100.0	100.0	100.0
normal walking	100.0	100.0	100.0	100.0	100.0	100.0	100.0	100.0	100.0	100.0
brisk walking	100.0	100.0	100.0	100.0	100.0	100.0	100.0	100.0	100.0	100.0
jogging	100.0	100.0	100.0	100.0	100.0	100.0	100.0	100.0	100.0	100.0
Total discrimination	99.8%	99.1%	99.6%	99.1%	99.4%	98.6%	99.1%	98.6%	98.9%	98.6%

*shows the excellent cut-off value of children to discriminate between locomotive and nonlocomotive activity in this study.

# shows the cut-off value of adults to discriminate between locomotive and nonlocomotive activity which was proposed in our previous study [20].

### Nonlocomotive Activities Equation (Development Group: n = 48)

METs = 0.013×synthetic acceleration +1.235, R^2^ = 0.752, RSME = 0.694 (standard equation)

### Locomotive Activities Equation (Development Group: n = 48)

METs = 0.005×synthetic acceleration +0.878, R^2^ = 0.884, RMSE = 0.651 (standard equation)

Next, we examined the cross-validation of the new calibration model in the cross-validation group (n = 20). The rate of correct discrimination was 99.1% when the cut-off value of 1.12 was used to discriminate PAs in cross-validation group. The absolute differences were less than or equal to 0.50 METs, excluding climbing down and up (Table 4). Finally, we proposed an equation from the data of all participants (the development group combined with the cross-validation group).

**Table 4:** Absolute and percentage differences between measured and predicted METs from each equation model for nonlocomotive and locomotive activities in the cross-validation group (n = 20).

	Predicted METs		Measured METs		Absolute difference		% difference		*P* value
	Mean	SD	Mean	SD	Mean	SD	Mean	SD	
***Nonlocomotive***									
desk work	1.34	0.06	1.15	0.13	0.19	0.13	17.5	15.0	<0.01
Nintendo DS	1.30	0.03	1.11	0.09	0.18	0.10	17.9	9.1	<0.01
sweeping up	3.29	0.72	3.15	0.73	0.14	0.46	5.8	14.6	NS
clearing away	2.77	0.40	3.01	0.58	−0.25	0.42	−6.5	12.8	NS
washing the floor	3.91	0.40	4.41	0.69	−0.50	0.79	−9.0	18.9	<0.01
throwing a ball	4.26	0.78	3.76	0.82	0.48	0.45	14.9	13.4	<0.05
***Locomotive***									
climbing down	2.88	0.27	2.26	0.28	0.58	0.41	29.1	20.5	<0.01
climbing up	2.20	0.20	5.28	0.69	−3.08	0.61	−58.0	4.7	<0.01
normal walking	2.54	0.21	2.58	0.24	−0.04	0.36	−0.6	13.8	NS
brisk walking	3.21	0.25	3.16	0.25	0.05	0.36	2.1	11.3	NS
Jogging	6.44	0.48	6.20	0.77	0.23	0.83	5.2	14.7	NS

*P*<0.05 and <0.01 show that mean values were significantly different compared with measured METs.

METs; metabolic equivalents, SD; standard deviation, NS; not significant.

### Nonlocomotive Activities Equation (Total Participants: n = 68)

METs = 0.013×synthetic acceleration +1.220, R^2^ = 0.772, RMSE = 0.664 (standard equation)

### Locomotive Activities Equation (Total Participants: n = 68)

METs = 0.005×synthetic acceleration +0.944, R^2^ = 0.880, RMSE = 0.639 (standard equation)

Furthermore, the inclusion of weight, chronological age and sex significantly improved the prediction accuracy of the locomotive equation. Chronological age and sex were significant variables in the nonlocomotive equation. However, the interaction term between chronological age and sex was not significant in either equation (Table 5).

**Table 5:** Effect of weight, age and sex on predictive ability by multiple regression analysis.

Independent variable	Intercept	Regression coefficient	*P* value	Adjusted R^2^	RMSE
***Nonlocomotive***					
** Model 1**					
synthetic acceleration (mg)	1.220	0.013	<0.001	0.772	0.664
** Model 2**					
synthetic acceleration (mg)	−0.537	0.013	<0.001	0.816	0.596
weight			NS		
age		0.170	<0.001		
sex (boys:0, girls:1)		0.076	<0.05		
***Locomotive***					
** Model 1**					
synthetic acceleration (mg)	0.944	0.005	<0.001	0.880	0.639
** Model 2**					
synthetic acceleration (mg)	−0.925	0.005	<0.001	0.925	0.508
weight		0.032	<0.001		
age		0.085	<0.01		
sex (boys:0, girls:1)		0.092	<0.05		

RMSE; root mean square error, NS; not significant.

We compared each MET value obtained from the standard equation and the multiple regression equation with the METs measured during each PA (Table 6). Although a slight improvement in the predictive equation (R^2^ and RMSE) was observed in both nonlocomotive and locomotive activities, we could not find significant improvements in the predictive ability for each activity (Table 6).

**Table 6:** Comparison between predicted METs from each equation and measured METs (n = 68).

	Standard equation				Multiple regression equation				Measured METs		ANOVA
	Predicted METs		Difference*		Predicted METs		Difference*				
	Mean	SD	Mean	SD	Mean	SD	Mean	SD	Mean	SD	
***Nonlocomotive***											
desk work	1.32	0.06	0.17	0.11	1.32	0.29	0.17	0.28	1.15	0.10	St, Mu>Me
Nintendo DS	1.30	0.04	0.18	0.10	1.30	0.28	0.28	0.27	1.12	0.09	St, Mu>Me
sweeping up	3.23	0.58	0.25	0.55	3.21	0.57	0.24	0.41	2.97	0.57	St, Mu>Me
clearing away	2.81	0.41	−0.23	0.58	2.80	0.46	−0.25	0.48	3.05	0.60	Me>St, Mu
washing the floor	3.98	0.48	−0.65	0.88	3.96	0.46	−0.66	0.70	4.62	0.78	Me>St, Mu
throwing a ball	4.20	0.80	0.53	0.60	4.19	0.80	0.53	0.47	3.69	0.65	Mu, St>Me
***Locomotive***											
climbing down	2.96	0.35	0.67	0.42	2.92	0.48	0.64	0.42	2.31	0.26	S, Mu>Me
climbing up	2.39	0.33	−2.91	0.74	2.39	0.52	−2.94	0.57	5.30	0.69	Me>S, Mu
normal walking	2.66	0.21	0.10	0.34	2.64	0.44	0.05	0.33	2.56	0.27	NS
brisk walking	3.34	0.34	0.16	0.36	3.29	0.45	0.09	0.32	3.16	0.33	S>Me
Jogging	6.69	0.59	0.26	0.99	6.46	0.76	0.02	0.75	6.43	1.04	NS

*Mean and SD mean the difference between predicted METs from each equation and meausred METs.

METs; metabolic equivalents, SD; standard deviation, ANOVA; analysis of variance, NS; not significant; St, standard equation; Mu, multiple regression equation; Me, measured.

>(a sign of inequality) means a significant difference among equations.

The predicted values from standard equation for washing the floor (−0.65±0.88; METs, −11.4±18.8%) and climbing up (−2.91±0.74; METs, −54.2±9.1%) were significantly underestimated compared with the measured values. The predicted values of desk work (0.17±0.11; METs, 15.7±11.2%), Nintendo DS (0.18±0.10, METs, 17.1±10.4%), throwing a ball (0.53±0.60, METs, 15.7±18.1%) and climbing down (0.67±0.42; METs, 30.9±20.2%) were significantly overestimated. However, we did not observe significant differences between the predicted values and the measured values for sweeping up, clearing away, or brisk walking and jogging (Table 6).

**Figure 3:**
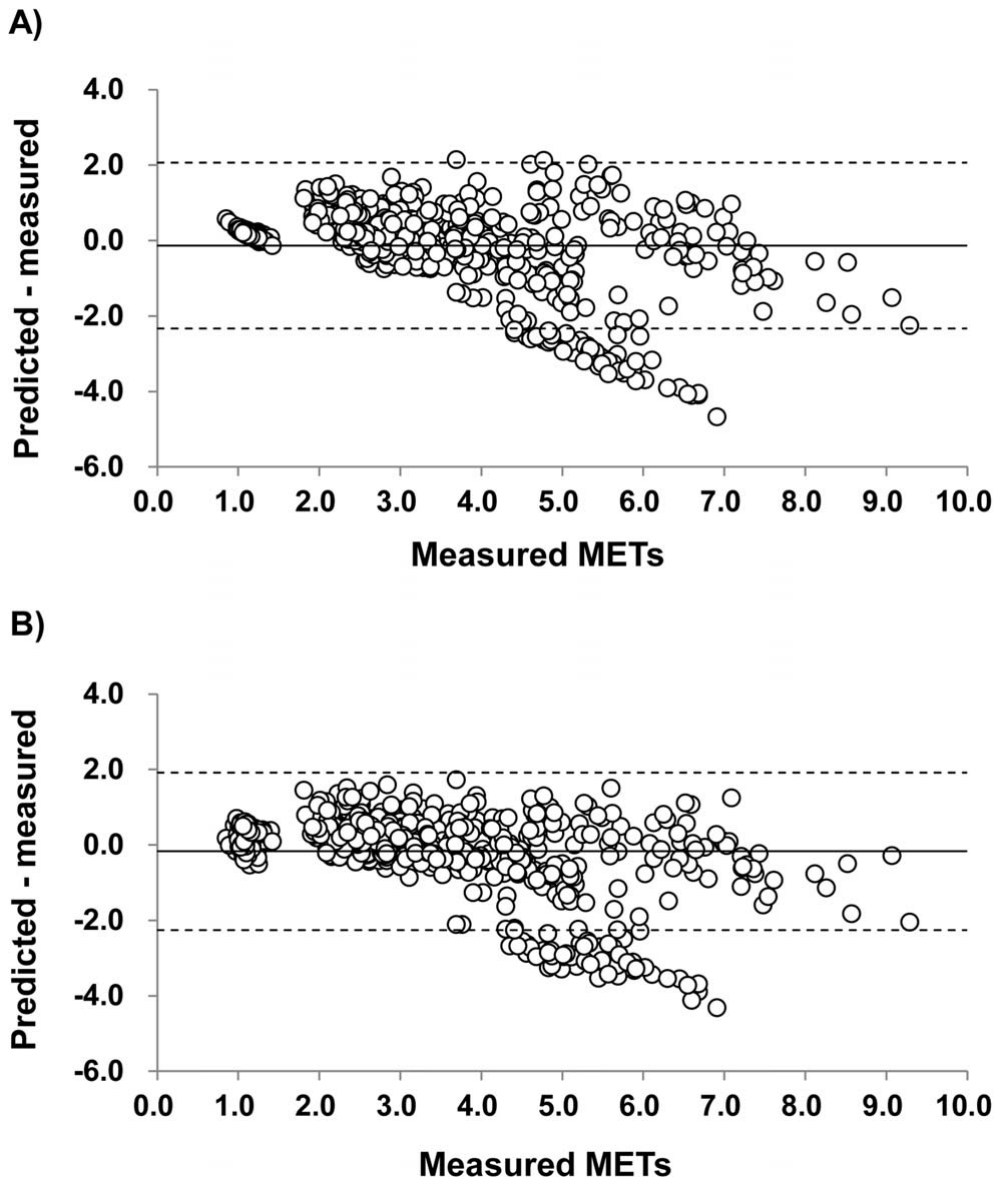
Differences between predicted and measured METs from each equation by Bland and Altman plot analysis. The solid line represents mean differences between measured and predicted values. The 2 dashed lines represent the upper and lower limits of agreement, calculated as mean difference ±2 SD. Upper figure (A) and lower figure (B) shows the standard equation’s plots and the multiple regression equation’s plots, respectively.

In addition, the differences between the measured METs and the predicted METs from each equation were determined by Bland-Altman analysis (Figure 3). The standard equation showed a mean difference of −0.13 METs and limits of agreement (±2 SD) from +2.06 to −2.33 METs. The multiple regression equation showed a mean difference of −0.17 METs and limits of agreement (±2 SD) from +1.91 to −2.26 METs.

## Discussion

Many studies have reported that accelerometers are excellent devices for the estimation of locomotive activities, such as walking and jogging on a treadmill or on the ground [29], [30]. However, recently, several studies reported that it was difficult to estimate PA intensity for children using the existing predictive model [10], [11], [14]–[17], because the habitual PA behaviors of children are more complex and poorer economically [31], [32], and they change more frequently than those of adults [4], [5]. To be precise, a predictive equation based on locomotive activities led to an underestimation of PA intensity during nonlocomotive activities, such as household tasks [11]. This might mean that discriminating locomotive from nonlocomotive activities contributes to the estimation accuracy of PA intensity in children. Therefore, in the current study, we tried to examine whether the GRPACA, which was developed in our calibration model for adults, is able to discriminate various PAs in children, and to prove that this discrimination method improves the estimation accuracy of the prediction model for children using an accelerometer.

Our first key finding was that it might be possible to apply the discrimination procedures developed in adults to any participant with various activity components and patterns. In our previous study, we found that the percentage of correct discrimination with the GRPACA in adults was remarkable, 98.7%, when the ratio of USA/FSA was 1.16 [21]. In the present study, when the threshold of discrimination, which was similar to that in the previous study, was 1.12, the rate of correct discrimination was excellent, at 99.1% on average (Figure 1, Table 3). As the discrimination method that used the coefficient of variation in a previous study was 97% for locomotive activities and 89.5% for nonlocomotive activities [17], our discrimination procedure had a better rate of correct discrimination. It follows that our specific calibration model could evaluate the PA intensity of children with an estimation accuracy of a mean difference of −0.13 METs and limits of agreement (±2 SD) from +2.06 to −2.33 METs, similar to the success we obtained with the adult model in our previous study for adults [20], [21]. This finding was supported by a strong linear relationship in the two prediction formulas and a cross-validation trial with another group of children (Table 4). These results suggested that our specific model, established according to the procedure of the adult model, was well suited to evaluate the PA of children.

We did not simultaneously compare our device with major devices, such as ActiGraph. However, our calibration procedures followed the procedures used in several calibration studies [11]–[17], which enabled comparison of the results in the present study with previous studies that used a common device. For example, a proposed single equation using a common device such as ActiGraph, Actical or RT3 provides average prediction errors of more than about 20% for nonlocomotive activities, calculated from average published values like VO_2_ (ml/kg^0.75^/min), activity energy expenditure (kcal/kg/min) and METs [14], [33], [34], [35]. Moreover, when our model was compared with the 2 RM with ActiGraph proposed recently, the differences between the predicted METs and the measured METs in the current study were slightly smaller than those of the previous study [17]. To be more precise, the differences with ActiGraph for vigorous intensity PAs, such as sportwall and running, were −1.8 to METs and −1.1 METs [17], respectively, while the differences with our model were 0.23 METs for similar-intensity PAs like jogging. Furthermore, the difference with our model, which was within 0.50 METs for all PAs including sedentary to vigorous intensities, except for climbing up and down, was slightly smaller than in the previous study (within 0.6 METs) [17]. Actually, another study also indicated that the 2 RM with ActiGraph had a disadvantage for sedentary and high intensity PAs [36]. In the current study, although there were significant differences between the measured METs and the predicted values from standard equations in washing the floor, throwing a ball, and climbing down and climbing up, mean differences compared to the measured METs in overall activities were small (−0.13±1.09 METs). Mean differences between the predicted METs and the measured METs only in sedentary behaviors to light intensity PAs (<3.0 METs), which consumed the highest percentage of time per day [37], were still minimal (−0.20±0.33 METs) in the current study.

The finding that our procedure could lead to comparable estimation accuracy in both nonlocomotive and locomotive activities was also significant. The cause might depend on the fact that our model could assess upper-body activities such as sweeping up, clearing away, and throwing a ball accurately. Oshima et al. [21] indicated that when the acceleration sensor was attached to the waist of the individual, the USA/FSA ratio reflected dynamic changes in body posture. The waist is not in the upper body, but the inclination of the upper body accompanies that of the waist in most instances. Therefore, the gravitational acceleration signal at the waist reflects postural changes of the upper body during nonlocomotive activities, like household activities, to some degree.

In the present study, we also found that the adjusted determination coefficient (R^2^) and the root mean square error (RMSE) were slightly better when weight, chronological age, and sex were added as independent variables into the standard predictive equations when combining the development group with the cross-validation group (Table 5). However, we did not observe significant differences between the multiple regression equation and the standard equation (not controlled) when looking at the average prediction error for each activity (Table 6). As this would mean that the integrated acceleration from the three dimensions associated with a child’s motion includes the effects of biological factors, it might not be necessary to control for weight, age, and sex, similar to several other calibration studies [15], [16].

### Limitations

Given the limitations of this study, we must be very careful when interpreting our results. We cannot conclude that this predictive model is superior to previous calibration models proposed using common devices, because we did not directly compare our model to other models using the same experimental conditions (i.e. device, ethnic group, targeted activities, and calculation of energy expenditure in the resting state). To truly prove superiority, it would be necessary to compare the different methods under free-living conditions. Furthermore, in the future, we must determine whether our developed model is applicable for estimating PAs not including calibration tasks, because the predictive accuracy of the existing model is significantly reduced when applied to non-calibration activities [17], [35].

## Conclusions

The results of this study indicate that a specific calibration model that discriminates between nonlocomotive and locomotive activities for children can be useful to evaluate the sedentary to vigorous PAs of both nonlocomotive and locomotive activities. One of the main reasons why the differences between predicted and measured METs with our model were smaller than those reported in previous calibration studies using common devices may be the model’s high rate of correct discrimination between locomotive and nonlocomotive activities.
